# The relationship between green space and myopia in children and adolescents: a systematic review and meta-analysis

**DOI:** 10.3389/fpubh.2026.1712259

**Published:** 2026-04-01

**Authors:** Junjie Wang, Gang Sun, Cuimei Shen, Houwei Zhu, Zhanyang He

**Affiliations:** 1 College of Physical Education and Health Sciences, Zhejiang Normal University, Jinhua, China; 2 Department of Business Administration, Shanghai University of Finance and Economics Zhejiang College, Jinhua, China

**Keywords:** adolescents, children, environmental intervention, green space, myopia

## Abstract

**Objective:**

To investigate the effect of green space exposure on the risk of myopia in children and adolescents.

**Methods:**

Studies on the relationship between green space and myopia in children and adolescents published before October 1, 2024, were retrieved from five electronic databases: web of Science Core Collection, PubMed, EBSCOhost, ScienceDirect and Cochrane Library. A systematic review was conducted to summarize the relevant articles, followed by a meta-analysis.

**Results:**

A total of 11 studies were included in the systematic review, encompassing 2,224,332 participants aged 6–22 years. Of these, 6 studies were incorporated into the meta-analysis. Most studies indicated that increased green space is associated with a reduced risk of myopia. The Normalized Difference Vegetation Index (NDVI) showed a negative correlation with the prevalence of myopia in children and adolescents (cross-sectional studies: *OR* = 0.95, 95% CI: 0.94–0.96, *I*^2^= 37.2%, *P* = 0.158; cohort studies: *OR* = 0.91, 95% CI: 0.91–0.91, *I*^2^= 88.9%, *P* **<** 0.001). NDVI within school boundaries and a 500-meter buffer zone had a more significant effect on myopia prevention (within school boundaries: *OR* = 0.87, 95% CI: 0.77–0.99, *P* = 0.206; 500-meter buffer zone around schools: *OR* = 0.96, 95% CI: 0.94–0.97, *P* = 0.098; 1,000-meter buffer zone around schools: *OR* = 0.94, 95% CI: 0.92–0.97, *P* = 0.315) (within school boundaries: *OR* = 0.96, 95% CI: 0.94–0.98, *P* = 0.066; 500-meter buffer zone around schools: *OR* = 0.90, 95% CI: 0.90–0.91, *P* = 0.053).

**Conclusion:**

Increased exposure to green spaces has a beneficial effect on reducing the risk of myopia in children and adolescents. For every 0.1 increase in the NDVI, the prevalence of myopia decreases. Furthermore, the prevention and control of myopia are more effective within school boundaries and in the surrounding 500-meter buffer zone. Therefore, it is recommended that schools and relevant government authorities focus on enhancing vegetation coverage within school grounds and around areas where students frequently engage in activities, thereby providing stronger environmental support for myopia prevention in children and adolescents. However, potential publication bias cannot be excluded given the limited number of included studies; therefore, the pooled estimates should be interpreted with caution.

**Systematic review registration:**

https://www.crd.york.ac.uk/PROSPERO/view/CRD420251153654, PROSPERO: CRD420251153654.

## 1 Introduction

Myopia is a refractive error in which light entering the eye through the refractive system does not directly focus on the retina, but instead focuses in front of it (1), typically caused by excessive elongation of the axial length of the eye, especially through the extension of the vitreous cavity (2). Over the past few decades, the global incidence of myopia has surged dramatically. In numerous countries, the prevalence of myopia surpasses 50% (3), and this proportion continues to increase, especially in the developed regions of East and Southeast Asia, Studies have found that in urban areas of these countries, 80%−90% of children and adolescents are affected by myopia, with 10%−20% potentially suffering from high myopia (1, 4). Meanwhile, according to the latest data released by China's National Health Commission in 2023, the overall myopia rate among Chinese children and adolescents is 52.7%, with the rate among high school students reaching as high as 80.5%. An increasing body of evidence suggests that the issue of myopia among children and adolescents is becoming more severe. The rising incidence, the trend toward younger ages, and the growing proportion of high myopia are all significantly increasing, This not only leads to separation anxiety in myopic patients, causing a sense of being unable to cope with the world, which in turn exacerbates anxiety, depression, and other negative emotions in children and adolescents (5), but it may also progress to high myopia, leading to serious ocular health complications (6, 7).

Engaging in activities in green spaces rich in vegetation is often considered to have a potential role in preventing myopia and slowing its progression. Green spaces are defined as open areas with vegetation, including places with “natural surfaces” or “natural environments,” such as gardens and nature reserves, as well as specific types of urban greenery, such as community parks, street trees, and greenbelts. To quantify and assess changes in exposure to green spaces, remote satellite imagery is commonly used. One commonly used method for assessing green space exposure is the Normalized Difference Vegetation Index (NDVI), which is calculated as NDVI = (NIR - R)/(NIR + R), where NIR refers to near-infrared and R denotes red (8). It should be noted that NDVI represents an area-level indicator of vegetation density rather than a direct measure of individual outdoor behavior or ocular light exposure. While higher NDVI is often correlated with increased opportunities for outdoor activity and greater ambient light availability, it does not capture how frequently or for how long children actually spend time outdoors, nor does it quantify individual-level light exposure (9). Therefore, NDVI should be interpreted as a proxy for environmental greenness rather than a direct behavioral or physiological exposure. While several other vegetation indices, such as the Green Ratio Vegetation Index (GRVI) [GRVI = NIR/G, where NIR is near infrared and G is green], the Soil Adjusted Vegetation Index (SAVI) [SAVI = ((NIR - R)/(NIR + R + 0.5)) ^*^ 1.5], and the Ratio Vegetation Index (RVI) [RVI = NIR/R], may more effectively capture vegetation signals across different seasons and regions (10).

Previous studies have indicated that exposure to green spaces can have both positive and negative effects. Empirical research suggests that interaction with green spaces offers benefits for both physical and mental health (11–14). The World Health Organization emphasizes the significant benefits of engaging in activities within green spaces (7), such as increasing physical activity, improving immune system function, and reducing obesity. Similarly, the presence of plants or other natural elements in urban areas can alleviate depression and stress levels, restoring individuals' mental wellbeing, and has positive effects on psychological health (10). It is noteworthy that the health benefits of green spaces are not unidirectional. Several studies have reported that, under certain circumstances, the expansion of vegetation may increase exposure to pollen and other aeroallergens (15), thereby elevating the risk of asthma or allergic diseases (16). This issue is directly relevant to the present study: if allergic or asthmatic symptoms lead children to spend less time outdoors or to avoid specific environments such as wooded or rural areas, the protective effect of green spaces on myopia—mediated through greater outdoor time and natural light exposure—may be attenuated. Therefore, when promoting the co-benefits of urban and school greening for visual health, the selection and spatial configuration of plant species should also take allergen control into account. Future studies are encouraged to consider allergy and asthma status as potential effect modifiers when examining the robustness of the association between green space exposure and myopia risk.

Over the past two decades, the causal relationship between green spaces and the health of children and adolescents has become a central focus in the evolving field of environmental health research. With the rapid pace of urbanization, the reduction of green spaces and the increasing incidence of myopia have prompted researchers to explore the connection between the two. Stanhope found a negative correlation between green spaces and excessive artificial light at night (ALAN), the latter of which has been identified as a factor contributing to myopia (17). At the same time, green spaces have been shown to increase exposure to natural light, stimulating the release of dopamine in the retina and regulating axial eye growth to prevent excessive elongation, while providing visual stimulation at various distances to help prevent myopia progression (18). Additionally, several epidemiological studies have documented an association between higher levels of green space and reduced blood pressure and psychological stress in children and adolescents (19–21), psychological stress can lead to tension in the muscles and tissues around the eyes, altering the shape of the eyeball and increasing the risk of myopia. Green spaces, by encouraging outdoor activities, increasing physical activity time, and reducing time spent on nearby tasks such as using electronic devices or reading, contribute to a reduction in myopia prevalence. However, some studies have found no statistically significant association between green spaces and myopia in children and adolescents (22). Although most studies employing remote sensing indicators such as the NDVI have reported an association between higher green space exposure and a lower risk of myopia, emerging evidence suggests that different green space assessment metrics are not interchangeable measures. These indicators capture distinct ecological and social dimensions, including vegetation coverage (NDVI/SAVI), availability of park resources (total or per capita park area), visible greenery or shadow indices (PSA), and perceived greenness based on questionnaires. Because these metrics differ in their sensitivity to accessibility, visual exposure, actual use, and seasonal variation, they may represent key sources of heterogeneity across observational studies. Systematically distinguishing and synthesizing findings based on different green space indicators and related influencing factors is essential not only for clarifying the inconsistencies in existing evidence, but also for providing actionable guidance for urban and school greening interventions—particularly in the design of environments surrounding schools and residential areas. Such work holds direct implications for public health policy and for mitigating the growing trend of myopia among children and adolescents. Therefore, in addition to quantifying the overall association between green space exposure and myopia, the present study explicitly compares different green space indicators as predefined analytical variables, using subgroup analyses and meta-regression to examine their roles as potential sources of heterogeneity.

Therefore, this study aims to systematically review the research on the relationship between green spaces and the incidence of myopia in children and adolescents both domestically and internationally, and to conduct a meta-analysis. The study will objectively evaluate and synthesize different methods and ranges for quantifying green space exposure, analyzing a wide range of evidence to clarify the relationship between green spaces and the onset of myopia in children and adolescents.

## 2 Methods

### 2.1 Study eligibility

This systematic review and meta-analysis, based on primary observational studies investigating the association between green space exposure and myopia, is registered in the International Prospective Register of Systematic Reviews (PROSPERO; registration number CRD420251153654). In addition and where applicable, the general guidelines of the Preferred Reporting Items for Systematic Reviews and Meta-Analysis (PRISMA) Statement were followed (23).

### 2.2 Literature retrieval

We conducted a systematic search across several electronic databases, including Web of Science Core Collection, PubMed, EBSCOhost, ScienceDirect and Cochrane Library, for publicly available studies examining the relationship between green spaces and myopia in children and adolescents. The search was limited to original research articles in both Chinese and English published before October 1, 2024. The search terms were based on a combination of three components: (1) myopia (Myopia OR Myopias OR Short-sight OR Short-sighted OR Short-sightedness OR Short sight OR Short sighted OR Near-sight OR Near-sighted OR Near-sightedness OR Nearsighted OR Near-sightedness OR Refractive Errors OR Refract); (2) green space (Green space OR Greenspace OR Greenness OR Green spaces OR Green areas OR Green OR Greenspaces); (3) children and adolescents (Children OR Child OR Adolescents OR Adolescent OR Youth OR Youths OR Teens OR Teenage OR Teenager OR Teenagers OR Student OR Students OR Adolescence OR pupil OR pupils OR Schoolchild OR juveniles). To ensure reproducibility, the full PubMed search strategy was provided as follows: (“green space”[Title/Abstract] OR “green spaces”[Title/Abstract] OR “greenspace”[Title/Abstract] OR “greenery”[Title/Abstract] OR “greenness”[Title/Abstract] OR “green”[Title/Abstract] OR “green areas”[Title/Abstract]) AND (“myopia”[Title/Abstract] OR “refractive error”[Title/Abstract] OR “refractive errors”[Title/Abstract] OR “near-sightedness”[Title/Abstract] OR “nearsightedness”[Title/Abstract] OR “near sightedness”[Title/Abstract] OR “axial length”[Title/Abstract] OR “spherical equivalent”[Title/Abstract]) AND (“child”[Title/Abstract] OR “children”[Title/Abstract] OR “adolescen^*^”[Title/Abstract] OR “adolescent”[Title/Abstract] OR “youth”[Title/Abstract] OR “youths”[Title/Abstract] OR “student”[Title/Abstract]). The search was carried out using a combination of subject terms and free-text terms, which were finalized after multiple pre-checks. If necessary, reference lists of previously included studies were traced. Retrieved articles were first screened by title and abstract, then full texts were extracted for evaluation. The search process was independently conducted by two researchers, and disagreements were resolved by consulting a third researcher.

### 2.3 Literature screening

According to the PICOS guidelines, the inclusion criteria for the literature are as follows: (1) P: the study subjects are children and adolescents, and relevant studies are included; (2) I: studies that examine green space as an exposure factor and its relationship with myopia are included; (3) C: studies that compare myopic patients with healthy controls; (4) O: the reported outcomes include the relationship between green spaces and myopia, such as positive or negative effects; (5) S: observational studies (cross-sectional studies, cohort studies, case-control studies) and intervention studies are included; (6) Only complete studies with full-length publications are included; (7) Only peer-reviewed articles from English and Chinese journals are included; (8) To meet the requirements for effect size merging in meta-analysis, the included studies must provide the effect size of the relationship between green space and myopia, along with its 95% confidence interval (CI), or sufficient data to calculate these values.

The exclusion criteria for the literature were as follows: (1) Exclusion of studies involving infants, adults, animals, or special populations such as professional athletes or individuals with disabilities; (2) Exclusion of studies on topics other than the relationship between green spaces and myopia; (3) Exclusion of reviews, commentaries, case reports, case series, qualitative research, etc.; (4) Exclusion of studies for which the full text could not be obtained; (5) Exclusion of non-English or non-Chinese literature, unpublished studies, conference abstracts, and theses.

### 2.4 Data extraction and quality assessment

Two researchers independently performed data extraction and quality assessment of the included studies, conducting two rounds of evaluation. In cases of discrepancies between the results of the two rounds, the opinion of a third researcher was sought, and a re-evaluation was conducted until consensus was reached. The types of extracted data included: first author's name, year of publication, basic characteristics of the study population (sample size, age range, gender, country or region), method of vision measurement, definition of myopia, method of green space measurement, method of obtaining myopia prevalence, outcome indicators, odds ratios (OR) and 95% confidence intervals (CI) from multivariate regression analysis of green space and myopia, or related data that can be calculated (such as relative risk (RR) or correlation coefficients), and covariates adjusted for in the multivariate regression model. Subsequently, we standardized all relative risk (RR) values to odds ratios (OR) using the following formula:


$$\begin{array}{l} OR=\frac{\left(1-P_{0}\right)\times RR}{\left(1-P_{0}\times RR\right)} \end{array}$$

Where *P*_0_ represents the disease incidence in the non-exposed group.

This review utilizes the research quality assessment criteria established by the Agency for Healthcare Research and Quality (AHRQ) to evaluate the quality of all included cross-sectional studies. The AHRQ Cross-Sectional Quality Assessment Criteria assess 11 aspects, including data sources, inclusion criteria, observation time, and subjective factors of the assessors. Each study is scored (“Yes” = 1 point, “No” or “Unclear” = 0 points). The maximum score is 11 points, with scores ≥ 8 indicating high quality, 4–7 points indicating moderate quality, and ≤ 3 points indicating low quality (24). The Newcastle-Ottawa Scale (NOS) is used to evaluate the quality of included cohort studies, scoring based on three aspects: selection of study population, comparability between groups, and outcome measurement. The maximum score is 9 points, with scores ≥ 8 indicating high quality, 5–7 points indicating moderate quality, and < 5 points indicating low quality (25).

### 2.5 Statistical analysis

Meta-analysis was conducted using Stata 16.0 software (StataCorp, 2019), with odds ratios (OR) and 95% confidence intervals (CI) as the effect size for combining the effect values (26). Before conducting the meta-analysis, a heterogeneity test was performed using the *I*^2^ statistic to evaluate the variability between the included studies. The *I*^2^ statistic quantifies the degree of heterogeneity, ranging from 0–100%, where higher values indicate greater heterogeneity among studies. If *P* > 0.1 and *I*^2^ ≤ 50%, it suggests minimal statistical heterogeneity between the studies, and a fixed effects model was used for the meta-analysis. If *P* < 0.1 and *I*^2^ > 50%, it indicates significant statistical heterogeneity, and a random effects model was employed. When substantial heterogeneity was detected (*I*^2^ > 50%), subgroup analyses and meta-regression were planned *a priori* to explore potential sources of heterogeneity (27). These analyses were conducted only when a sufficient number of studies (generally ≥10 studies per covariate, in accordance with established methodological recommendations) were available within each subgroup. When predefined subgroups contained only a single study, these subgroup-specific estimates were presented descriptively to reflect study-level characteristics rather than to draw inferential conclusions regarding sources of heterogeneity. This approach is consistent with methodological guidance indicating that subgroup analyses based on sparse data should be interpreted cautiously and are more appropriately presented in a descriptive manner (28). In addition, funnel plots were generated, and publication bias was assessed using Egger's tests when applicable (29).

## 3 Results

### 3.1 Literature selection process and results

**Figure 1:**
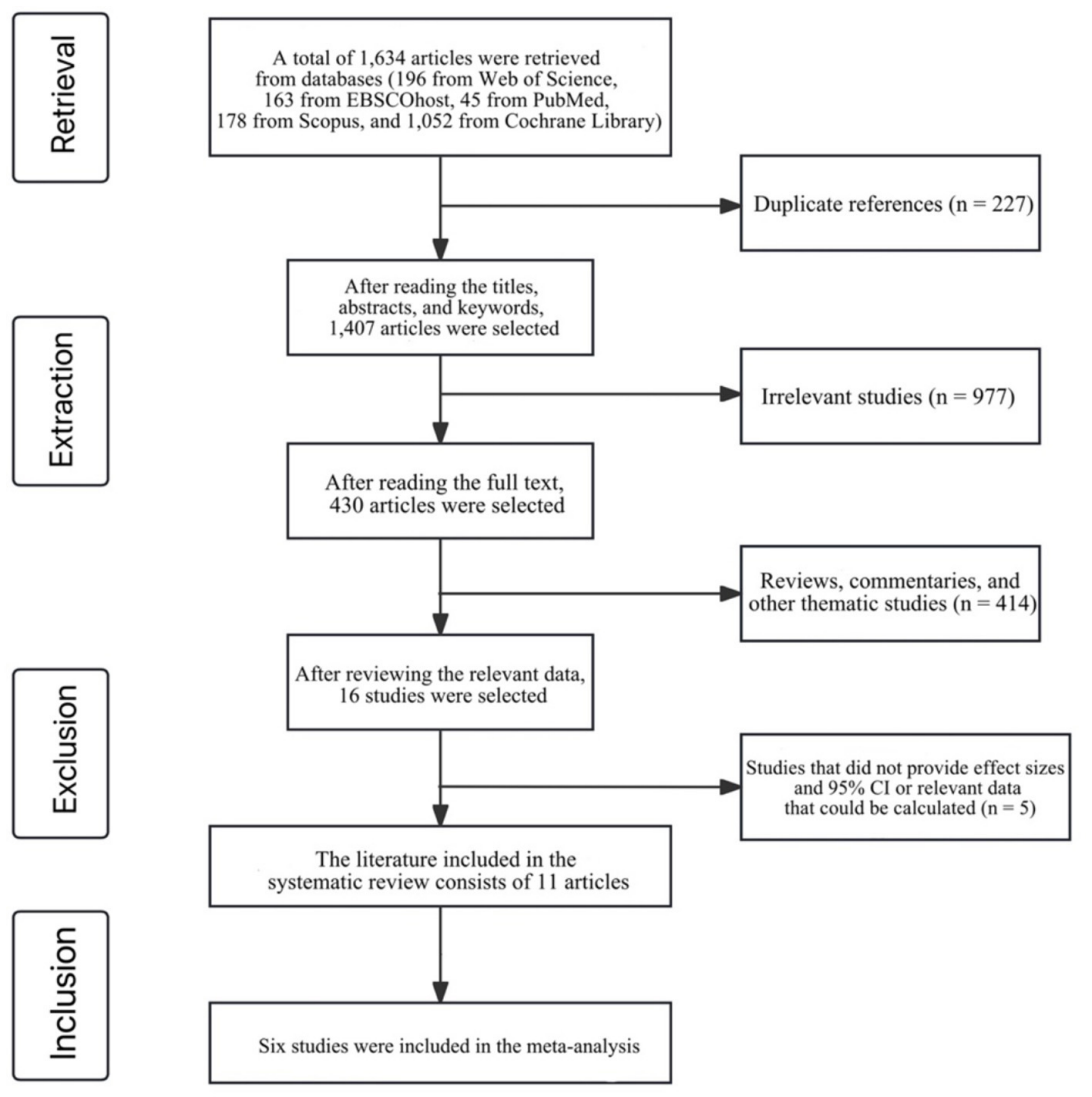
A flowchart summarizing all study assessment processes.

A total of 1,634 records were identified through subject-term searches across five major electronic databases, including Web of Science, EBSCOhost, PubMed, Scopus, and the Cochrane Library. All subsequent screening and selection procedures were conducted based on the studies retrieved from these sources. The retrieved articles were imported into the reference management software EndNote (Clarivate Analytics, 2023), and 227 duplicate articles were removed. Through reviewing titles, abstracts, and keywords, 977 unrelated studies were further excluded. Subsequently, the remaining 430 articles underwent full-text review, leading to the exclusion of 414 articles, including reviews, commentaries, and studies on other topics. Finally, for the remaining 16 articles, effect sizes and 95% confidence intervals (CI) or relevant data that could be calculated were sought. A total of 11 articles seven were included in the systematic review, with six articles included in the meta-analysis. These consisted of five longitudinal cohort studies, four cross-sectional studies, and 2 studies that combined both cross-sectional and longitudinal cohort designs (Figure 1).

### 3.2 Basic characteristics and quality assessment of studies included in the systematic review

A total of 11 studies published between 2017 and 2024 were included in this review (Table 1), encompassing a combined sample of approximately 2,224,332 children and adolescents, with individual study sizes ranging from 120–1,245,271 participants. The evidence base was predominantly derived from studies conducted in China (*n* = 9), complemented by one multinational study spanning 15 countries and one study conducted in Barcelona, Spain. The included studies comprised five longitudinal cohort studies, four cross-sectional studies, and two mixed-design investigations. Notably, eight studies (72.7%) enrolled more than 50,000 participants, providing substantial statistical power for detecting associations between green space exposure and myopia.

**Table 1:** Characteristics of studies included in review.

Serial number	Research literature	Participant information	Study design	Green space measurement	Quantitative data supporting results	Results
1	Yang et al. (31)	138,735 individuals, aged 6–9 (China)	Cohort study	NDVI within 500m buffer around schools	1.1-unit increase in the green space morphology index: 1.7% decrease in school-level myopia prevalence change (95% CI: −2.7–−0.6; *P* = 0.002) 2. 1-unit increase in the green space morphology index: 9.8% reduction in individual incident myopia risk (95% CI: 4.1–15.1; *P* < .001)	Higher green space proportion, aggregation, and connectivity associated with slower myopia increase.
2	Yang et al. (32)	159,895 individuals, aged 6–9 (China)	Cohort Study	NDVI within schools	1.0.1 increase in green space exposure: 3.6% reduction in school-level myopia prevalence over 2 years (95% CI: 1.8%−5.5%; *P* = 0.0001) 2. 0.1 increase in green space exposure: 19.8% reduction in individual myopia risk (AOR: 0.802, 95% CI: 0.724–0.889; *P* < 0.0001)	Green space exposure negatively correlated with myopia risk.
3	Lu et al. (36)	1,245,271 individuals, ages 8–15 (China)	Cohort Study	NDVI in 250m, 500m, 1,000m buffers around schools	1. NDVI 0.1 increase (250m): 6.3% reduction in myopia risk (OR: 0.937, 95% CI: 0.915–0.960) 2. NDVI 0.1 increase (500m): 7.7% reduction in myopia risk (OR: 0.923, 95% CI: 0.900–0.946) 3. NDVI 0.1 increase (1,000m): 8.7% reduction in myopia risk (OR: 0.913, 95% CI: 0.889–0.937)	Green space exposure slows myopia progression.
4	Huang et al. (34)	53,575 individuals, ages 4–6 (China)	Cohort study	NDVI within 100m, 250m, 500m buffers around homes	Neighborhood greenness within 100m increase: 38% reduction in myopia risk (AOR: 0.62, 95% CI: 0.38–0.99)	Residential greenery associated with reduced risk of myopia and astigmatism.
5	Bao et al. (35)	286,801 individuals, ages 7–9 (China)	Cohort Study	NDVI within 100m, 300m, 500m buffers around home and school	NDVI 300m 0.1 increase: 7% reduction in visual impairment risk (HR: 0.93, 95% CI: 0.92–0.94)	Green space exposure beneficial for visual development.
6	Zhang et al. (22)	120 individuals, ages 15–17 (China)	Cross-Sectional study	PSA and PGA within 500m buffer	PSA >20%: protective effect against moderate and high myopia, OR below threshold increases myopia risk	Higher shaded areas correlated with less myopia, but green space area showed no significant effect.
7	Zhang et al. (30)	13,380 individuals, ages 11–21 (China)	Cross-Sectional Study	NDVI in 500m, 1,000m buffers around schools	500m buffer NDVI 0.1 increase: 15% reduction in school-level myopia prevalence (OR: 0.85, 95% CI: 0.74–0.98)	Significant effect of school-zone NDVI on myopia risk.
8	Yang et al. (33)	61,995 individuals, ages 6–18 (China)	Cross-Sectional Study	NDVI and SAVI within 500m, 1,000m buffers around schools	NDVI 500m interquartile range increase: 5% reduction in visual impairment odds (OR: 0.95, 95% CI: 0.93–0.97).	Greener school surroundings lower risk of visual impairments.
9	Kai et al. (39)	261,833 individuals, ages 7–22 (China)	Cross-Sectional study	Park green space area per 10,000 people	Per capita green space increase: negative correlation with visual acuity loss (coefficient: −0.145; *P* < 0.001)	Larger park areas associated with reduced visual acuity issues.
10	Peng et al. (38)	15 countries, ages 15–19 (15 countries)	Cross-Sectional & cohort study	NDVI within 30 km radius around towns	Myopia prevalence significantly increases when green space is < -0.2, with a weaker effect for values >-0.1	Non-linear relationship: lower green space = higher myopia prevalence.
11	Dadvand et al. (37)	2,727 individuals, ages 7–10 (Barcelona)	Cross-Sectional Study	NDVI within 100m, 250m, 500m buffers around homes and schools	1. IQR increase in home greenness: 23% reduction in spectacles use (95% CI: 4%−39%) 2. IQR increase in school greenness: 34% reduction in spectacles use (95% CI: 2%−55%)	More green spaces associated with reduced need for glasses in children.

#### 3.2.1 Heterogeneity in green space exposure and myopia outcome measurements

Substantial heterogeneity was observed across the included studies in both exposure assessment and outcome measurement. This variability represents a key source of between-study differences and is essential for interpreting synthesized effect estimates.

Exposure indicators. The Normalized Difference Vegetation Index (NDVI) was the most commonly used metric for quantifying green space exposure (*n* = 9). Other studies employed alternative indicators, including the Soil-Adjusted Vegetation Index (SAVI), the proportion of green area (PGA), perceived or visible greenness (PSA), and per-capita park green space area (see the “Green Space Measurement” column in Table 1). Several cohort and cross-sectional studies reported risk variations associated with each 0.1-unit increase in NDVI or provided equivalent regression coefficients. For example, multiple studies indicated that a 0.1 increase in NDVI was associated with an approximately 5%−10% reduction in myopia risk. These quantitative parameters form the core evidence base supporting the synthesized conclusions of this review.

Spatial scales. The spatial resolution of exposure assessment varied widely across studies, ranging from school boundary–level measures to 100 m, 250 m, 300 m, 500 m, and 1,000 m buffers, as well as broader urban-scale measurements up to 30 km. Several studies demonstrated systematic scale-dependent effects, with stronger associations typically observed for green space exposure within school boundaries or 500 m buffers. Table 1 entries for Yang et al., Lu et al., Yang et al., and Bao et al. provide numerical evidence (e.g., percentage reductions and OR estimates) supporting this pattern. These findings suggest that green space within children's immediate daily activity spaces—particularly around schools and commuting routes—may exert more direct influence on visual development than distal greenness.

Myopia definitions and assessment methods. Outcome measurements also varied substantially. Definitions included spherical equivalent (SE ≤ −0.5 D, *n* = 5; SE ≤ −1.0 D, *n* = 1), uncorrected visual acuity (UCVA ≤ 4.9 or visual acuity < 6/6, *n* = 3), equivalent spherical lens (ESL ≤ −3.0 D, *n* = 1), and the use of corrective glasses as a proxy (*n* = 1). Measurement approaches included self-reported questionnaires (*n* = 3), visual acuity charts (*n* = 4), and autorefractors (*n* = 3). This lack of uniformity in myopia definitions and measurement techniques affects baseline prevalence estimates and limits comparability of effect sizes. Therefore, outcome variability was treated as a predefined source of heterogeneity in subsequent synthesis and subgroup analyses.

### 3.3 Quality assessment

According to the quality assessment tools applied in this review, eight studies were rated as high quality and three as moderate quality (Tables 2, 3). Overall, the high-quality studies were characterized by (1) clearly defined exposure and outcome measures, (2) adequate sample representativeness and, where applicable, sufficient follow-up, and (3) appropriate control of major confounders in multivariable models. These attributes strengthen the interpretability and generalizability of the evidence. However, variations across studies in exposure measurement scales, outcome definitions, behavioral mediators, and control of environmental co-exposures necessitate the use of random-effects models and prespecified subgroup analyses when synthesizing results in the presence of substantial heterogeneity.

**Table 2:** Quality assessment of included cohort studies.

Research literature	1A	1B	1C	1D	2	3A	3B	3C	Total score	Quality
Yang et al. (31)	1	1	1	1	1	1	1	1	8	High
Yang et al. (32)	1	1	1	1	2	1	1	0	8	High
Peng et al. (38)	1	1	1	1	1	1	1	0	7	Moderate
Lu et al. (36)	1	1	1	1	2	1	1	1	9	High
Huang et al. (34)	1	1	1	1	2	1	1	1	9	High
Dadvand et al. (37)	1	1	1	1	2	1	1	1	9	High
Bao et al. (35)	1	1	1	1	2	1	1	1	9	High

1. Selection of the study population: 1A indicates the representativeness of the exposed group (1 point); 1B describes the selection method for the unexposed group (1 point); 1C outlines the method for determining the exposure factor (1 point); 1D confirms that there were no outcome indicators to observe at the start of the study (1 point). 2. Comparability between groups: the comparability between the exposed and unexposed groups is considered in the design and statistical analysis (2 points). 3. Outcome measurement: 3A indicates whether the assessment of the outcome in the study is sufficient (1 point); 3B specifies whether the follow-up after the occurrence of the outcome is sufficiently long (1 point); 3C evaluates whether the follow-up for both the exposed and unexposed groups is adequate (1 point).

**Table 3:** Quality assessment of included cross-section studies.

Research literature	1	2	3	4	5	6	7	8	9	10	11	Total score	Quality
Zhang et al. (22)	1	1	1	1	1	1	1	0	0	1	0	8	High
Zhang et al. (30)	1	1	1	1	1	1	0	1	0	0	0	7	Moderate
Yang et al. (33)	1	1	1	1	1	1	1	1	0	1	0	9	High
Peng et al. (38)	1	1	1	1	1	1	0	0	0	0	0	6	Moderate
Dadvand et al. (37)	1	1	1	1	1	1	0	1	0	0	1	8	High
Kai et al. (39)	1	1	1	1	1	1	0	0	0	0	0	6	Moderate

1. Has the source of the data been clearly defined (e.g., survey, literature review); 2. Have the inclusion and exclusion criteria for the exposed and non-exposed groups been specified, or have previous publications been referenced; 3. Has the time period for patient identification been provided; 4. If the study population is not community-based, is the sample consecutive; 5. Is the assessment of subjective measures for patients separated from other objective indicators; 6. Have any quality assurance assessments been described (e.g., testing/retesting of primary outcomes); 7. Have the reasons for excluding patients been explained; 8. Have measures for evaluating and/or controlling confounding factors been described; 9. If applicable, has the handling of missing data in the analysis been explained; 10. Has the response rate of patients and the completeness of data collection been summarized; 11. If there is follow-up, has the percentage of missing data or incomplete follow-up results been clarified.

#### 3.3.1 Summary

Taken together, the available evidence demonstrates a high degree of consistency in the overall direction of associations. Most studies—particularly those employing NDVI as the primary exposure metric—consistently reported an inverse relationship between higher levels of green space exposure and lower risk of myopia. The quantitative estimates presented in Table 1, including the reported percentage reductions in risk per 0.1-unit increase in NDVI and the OR/HR values derived from multivariable models, provide direct empirical support for this directional conclusion.

However, substantial variability in exposure measurement approaches and in the definitions of myopia across studies may influence the absolute magnitude of effect estimates, and such differences should be taken into account when interpreting the findings. Notably, several studies in Table 1 indicate that green space exposure within school boundaries or within 500 m buffers tends to exhibit stronger protective effects, suggesting that children's and adolescents' daily activity spaces may represent critical zones for green space–based interventions.

### 3.4 The relationship between green spaces and the prevalence of myopia in children and adolescents

A structured synthesis of the 11 included studies reveals a generally consistent inverse association between green space exposure and myopia in children and adolescents, with the strength and statistical significance of this relationship varying according to the exposure metric, spatial scale, and analytical framework used in each study (Table 1). The vast majority of studies support a protective role of greenspace against myopia, although the effect strength appears to be modulated by the assessment methodology, spatial scale, and specific greenspace metrics employed. The core evidence indicates that greenspace coverage, as measured by the Normalized Difference Vegetation Index (NDVI), is a key indicator for assessing its protective effect. All six studies utilizing NDVI (30–35) consistently reported that higher NDVI values were associated with a reduced risk of myopia, with risk reduction estimates ranging between 8and 20%. Notably, this protective effect demonstrated a distinct spatial dependency: analyses based on NDVI within school boundaries and 500-meter buffers generally revealed stronger negative correlations, suggesting that the green environment within children's daily activity zones may confer more direct benefits for myopia prevention. Two studies (36, 37) further extended the evidence from prevalence outcomes to progression-related measures, reporting that higher surrounding greenness was associated with slower annual increases in myopic refractive error or lower likelihood of clinically meaningful visual acuity decline. These findings provide complementary behavioral and developmental insights, implying that green space may influence not only the onset but also the trajectory of myopic changes.

Not all relationships were strictly linear. One large-scale ecological study (38) identified a significant non-linear dose–response pattern, in which myopia prevalence increased sharply once greenness fell below a measurable threshold. This finding highlights the possibility that a minimum level of vegetation coverage is required before protective effects materialize, adding conceptual nuance to the interpretation of effect estimates. Beyond NDVI, the use of alternative green space metrics contributed to heterogeneity in results. Studies utilizing per capita park green space area (39) or visible/surface-based metrics such as PGA (proportion of green area) (22) reported mixed associations, with some detecting protective effects and others finding no significant relationship. These inconsistencies underscore that different greenness indicators capture distinct environmental dimensions—such as ecological biomass, accessibility, visual exposure, or landscape configuration—and are therefore not interchangeable. This inconsistency suggests that different greenspace metrics (e.g., coverage vs. area proportion) may capture distinct dimensions of the environment, thereby exerting differential effects on health outcomes. In summary, while there was some variation in how greenspace was measured and the strength of this link, the collective evidence robustly supports the conclusion that increased greenspace exposure, particularly through enhanced vegetation coverage in areas frequently used by children, such as schools and their immediate surroundings, represents a beneficial environmental strategy for reducing myopia risk in children and adolescents.

When considering contextual setting, school-based studies (*n* = 5) produced the most consistent findings, with nearly all reporting significant protective associations between increased on-campus or near-campus vegetation and reduced myopia risk. Residential-based studies showed greater heterogeneity, likely reflecting variability in population density, outdoor time behavior, parental supervision, and the mismatch between objectively measured greenness and children's actual exposure patterns. The single city-level study demonstrated a pronounced non-linear association between green space proportion and myopia prevalence, further suggesting that macro-level greenness may exert population-level effects, but these influences are conditioned by ecological thresholds and urban form. Despite variability in measurement methods and contextual frameworks, the collective evidence indicates a robust overall pattern: higher green space exposure—particularly vegetation coverage within school environments and their immediate surroundings—is associated with lower myopia prevalence and, in some cases, slower myopic progression. This spatial scale–dependent protective effect suggests that intervention strategies improving greenness in school-centric activity zones may yield the greatest preventive benefits for child and adolescent myopia. The observed heterogeneity across studies underscores the need for future research to adopt standardized greenness metrics, report exposure–response values transparently, and incorporate spatial behavior data to strengthen causal inference and reduce between-study variability.

#### 3.4.1 Meta-analysis of the relationship between the NDVI index and the prevalence of myopia in children and adolescents

Six studies assessed myopia as the outcome and reported NDVI measured in different buffer zones around schools. Among these studies, two were cross-sectional, three were cohort studies, and one employed a mixed cross-sectional/cohort design. We extracted the most fully adjusted effect estimate from each study and conducted separate syntheses for cross-sectional and cohort studies given their differing designs. First, heterogeneity tests were conducted separately for the cross-sectional and cohort studies. The results showed no significant heterogeneity between the cross-sectional studies (*I*^2^ = 37.2%, *P* = 0.158), so a fixed-effects model was used for analysis. However, there was considerable heterogeneity between the cohort studies (*I*^2^= 88.9%, *P* **<** 0.001), so a random-effects model was employed for the analysis.

**Figure 2:**
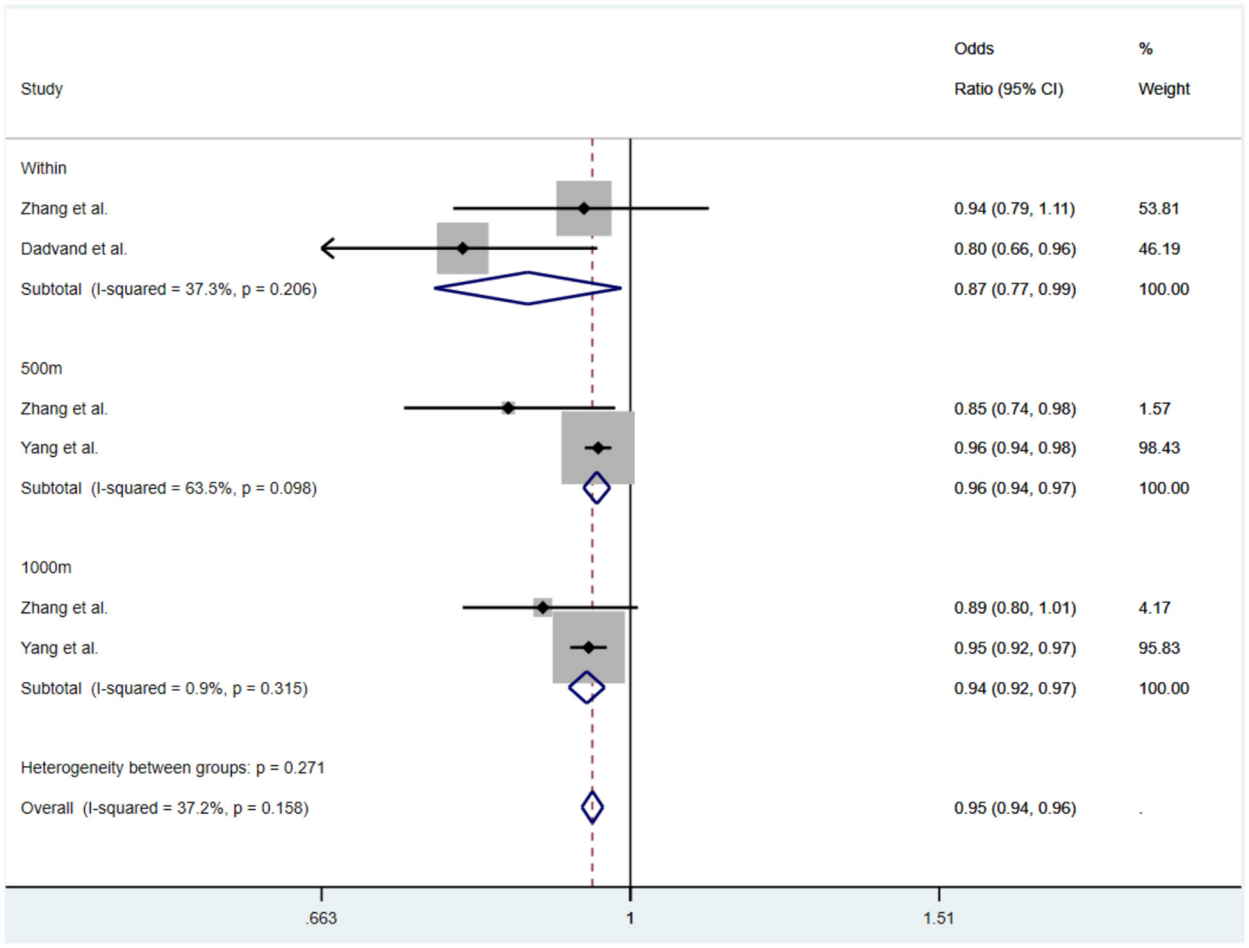
Subgroup analysis by cross-sectional studies of the association between green space exposure and myopia risk in children and adolescents.

The results of the cross-sectional study meta-analysis are shown in Figure 2. The combined OR is 0.95 (95% CI: 0.94, 0.96, *P* = 0.158). Additionally, the summary effect size within the school area in three studies showed an OR of 0.87 (95% CI: 0.77, 0.99, *I*^2^ = 37.3%, *P* = 0.206); within a 500m buffer zone centered on the school, the OR was 0.96 (95% CI: 0.94, 0.97, *I*^2^ = 63.5%, *P* = 0.098); within a 1,000m buffer zone centered on the school, the OR was 0.94 (95% CI: 0.92, 0.97, *I*^2^ = 0.9%, *P* = 0.315). The pooled estimate across these cross-sectional studies indicates an inverse association between NDVI and myopia: pooled *OR* = 0.95 (95% CI 0.94–0.96), i.e. the 95% confidence interval excludes 1, consistent with a statistically significant association in these studies. These results suggest a consistent inverse relationship between NDVI and myopia prevalence in children and adolescents; for example, an increase of 0.1 units in NDVI corresponds to a relative reduction in myopia prevalence in the order indicated by the pooled ORs above. Importantly, the effect on reducing myopia prevalence within the school area was more significant than that in the 500m and 1,000m buffer zones.

**Figure 3:**
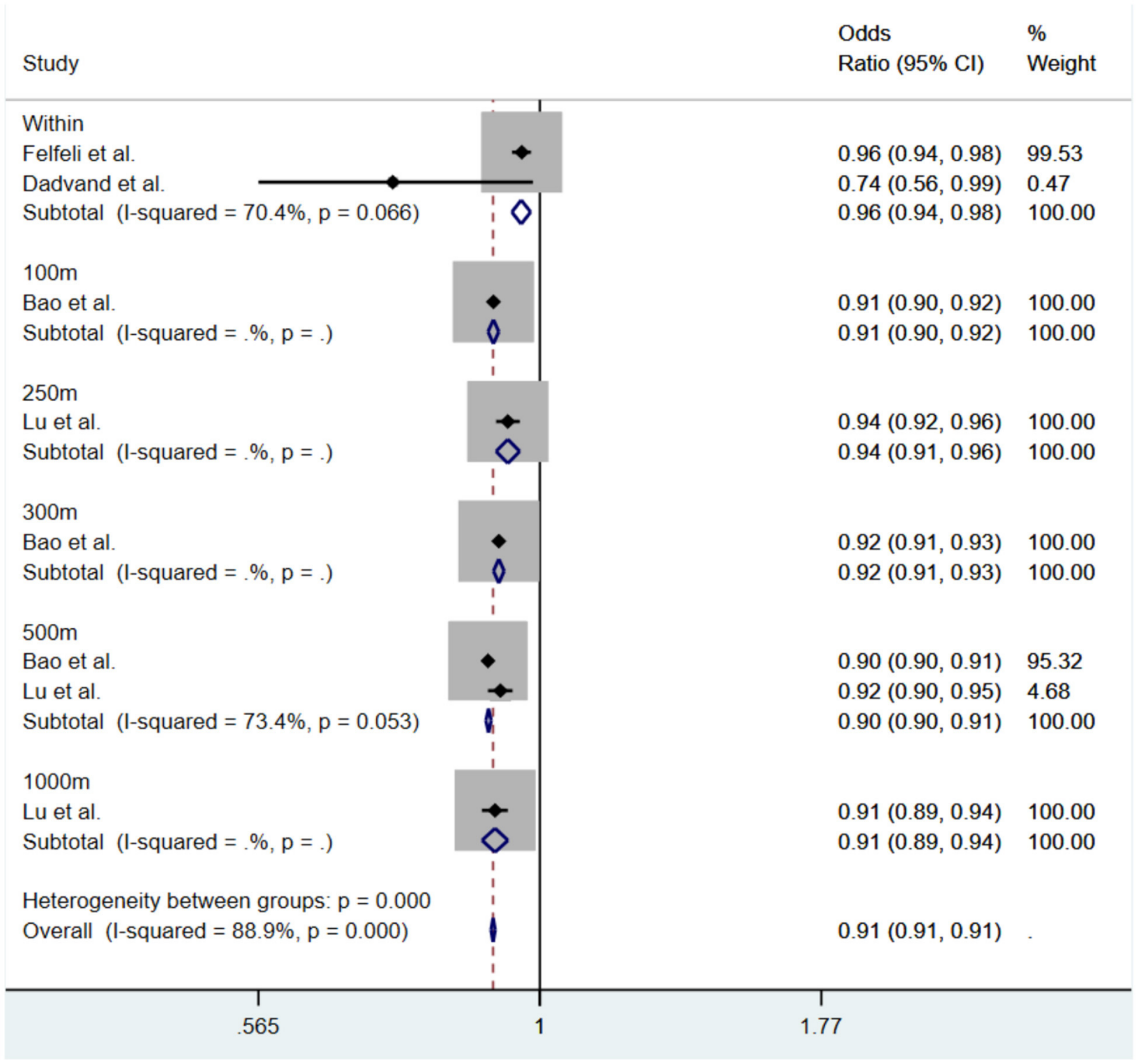
Subgroup analysis by cohort studies of the association between green space exposure and myopia risk in children and adolescents.

The results of the cohort study meta-analysis are shown in Figure 3. The combined OR is 0.91 (95% CI: 0.90, 0.91, P **<** 0.001). The pooled effect value within the school area is *OR* = 0.96 (95% CI: 0.94, 0.98, *I*^2^ = 70.4%, *P* = 0.066); within the 500m buffer zone centered around the school, the OR is 0.90 (95% CI: 0.90, 0.91, *I*^2^ = 73.4%, *P* = 0.053). The results indicate a negative correlation between NDVI and the myopia prevalence in children and adolescents, meaning that for every 0.1 increase in NDVI, the myopia prevalence decreases. The effect on reducing myopia prevalence is more pronounced within the 500m buffer zone around schools than within the school area itself. For cohort studies, although substantial heterogeneity was observed (*I*^2^ = 88.9%), further subgroup analyses or meta-regression were not performed because the number of eligible studies was insufficient to support reliable stratified analyses. For cross-sectional studies, subgroup analyses were conducted according to study region and exposure assessment methods.

**Figure 4:**
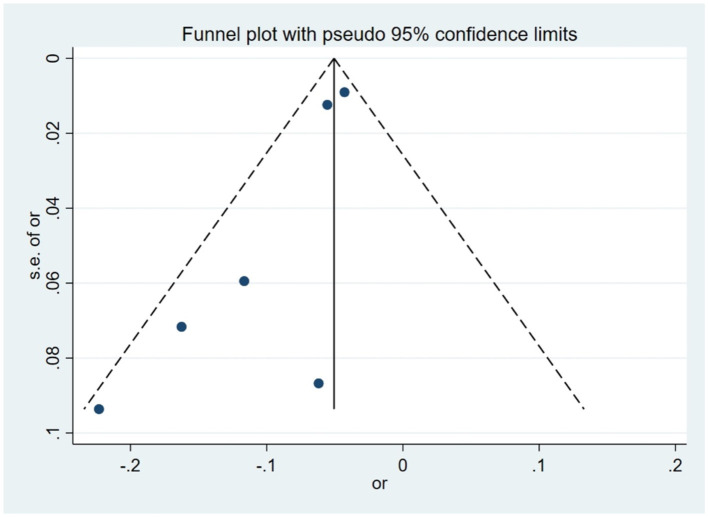
Funnel plot for publication bias in a cross-sectional study on the association between green space and myopia onset among children and adolescents.

**Figure 5:**
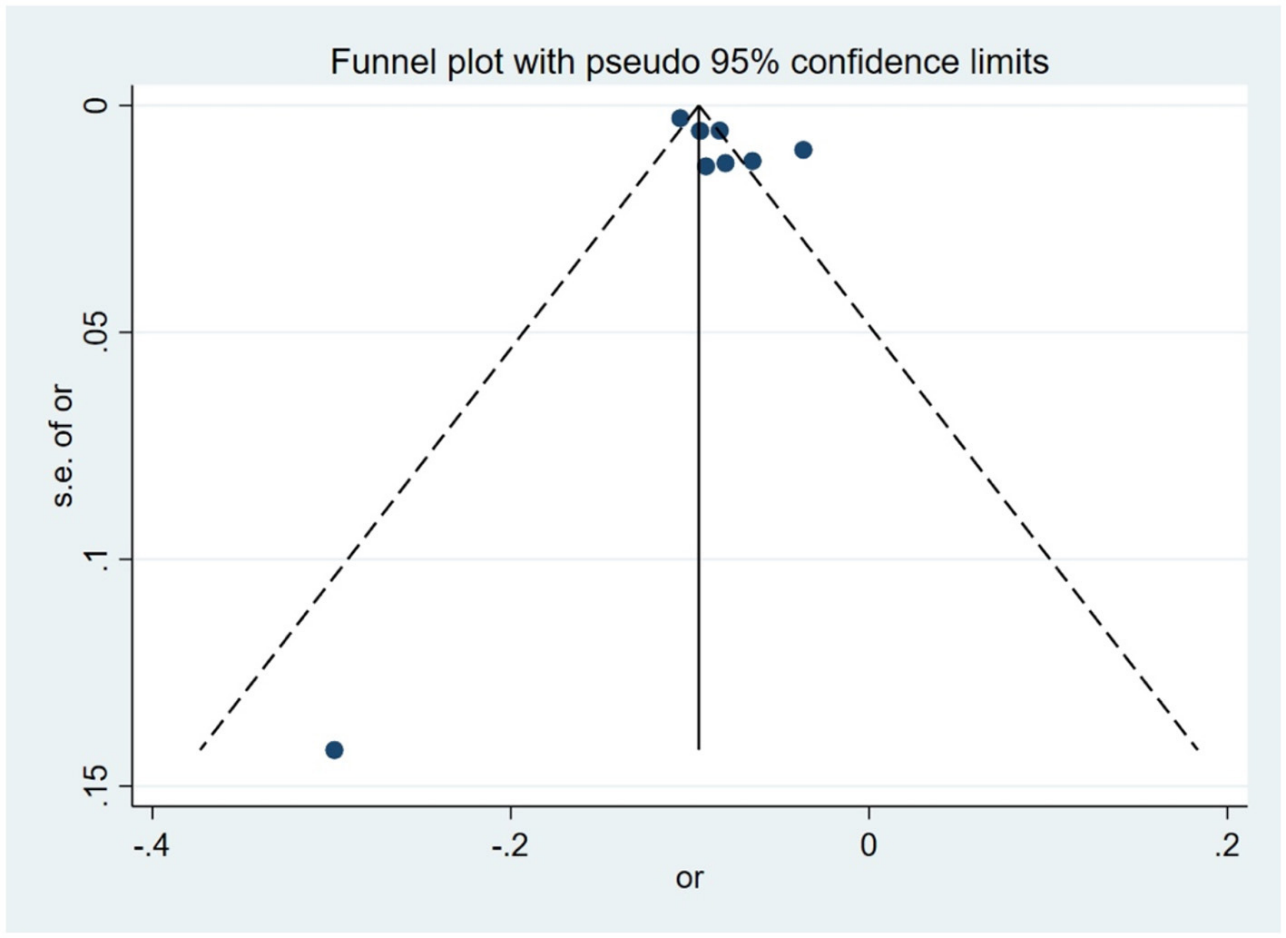
Funnel plot for publication bias in a cohort study on the association between green space and myopia onset among children and adolescents.

For the cross-sectional study (Figure 4), visual inspection of the funnel plot showed that most data points were distributed around the pooled effect estimate, although some degree of asymmetry was observed. Similarly, in the cohort study (Figure 5), although a few studies fall outside the 95% confidence limits, the overall distribution remains largely symmetrical, suggesting potential small-study effects or publication bias. Egger's test indicated evidence of small-study effects in the cross-sectional studies (*p* = 0.025), whereas no statistically significant publication bias was detected among cohort studies (*p* = 0.192) (Supplementary Table S1). However, given the limited number of studies included in several syntheses, the statistical power of funnel plot–based assessments and formal tests for publication bias is inherently constrained. Therefore, while no pronounced asymmetry was detected, the presence of publication bias cannot be fully excluded.

#### 3.4.2 Relationship between other covariates and the prevalence of myopia in children and adolescents

Evidence from the included studies demonstrates that the association between green space exposure and myopia is moderated by several individual and contextual covariates, although the overall strength of these moderating effects varies across studies. Gender was examined in three studies, which collectively suggest a sex-specific modification of green space effects. Two studies (30, 36) reported that higher NDVI values within 500–1,000 m buffers were associated with more pronounced reductions in myopia risk among girls, who also exhibited a consistently higher baseline prevalence of myopia. Another study (36), however, found the inverse pattern within a 300 m buffer, with boys demonstrating a stronger sensitivity to NDVI-related protective effects. These divergent findings indicate that gender may influence exposure–response patterns, but the current evidence base remains insufficient to draw definitive conclusions. Regional context was assessed in three studies, though only one (35) detected a significant interaction between suburban location and NDVI within a 500 m buffer, suggesting that the protective effect of greenness may vary across urban–suburban gradients. The remaining studies observed no meaningful association between geographic setting and myopia risk. Socioeconomic status (SES), included as a covariate in two studies, showed a more consistent pattern: children from lower SES areas exhibited a higher prevalence of myopia, and SES appeared to influence the magnitude of greenness-related protective effects, underscoring the interplay between environmental and socioeconomic determinants of visual health.

Screen time emerged as one of the most robust behavioral modifiers of the green space–myopia association. Two studies (31, 34) found that higher screen time attenuated the protective effects of NDVI, with negative correlations observed between NDVI and screen time in buffers ranging from 100 m to 500 m. These findings support the interpretation that green space may exert its benefits partly by promoting healthier behavioral patterns—particularly reducing prolonged near-work exposure—which in turn lowers myopia risk. Overall, while the moderating effects of gender and regional context remain mixed, the evidence consistently indicates that SES and screen time substantially condition the relationship between green space and myopia. These covariates should therefore be prioritized in future models assessing environmental contributions to visual health.

## 4 Discussion

Through a systematic review and meta-analysis of 11 studies (six studies included in the quantitative synthesis, total *N* = 2,224,332 participants), we found consistent evidence that greater green-space exposure around schools and residential areas is associated with lower myopia risk in children and adolescents. Quantitatively, cross-sectional studies pooled to an OR of 0.95 (95% CI 0.94–0.96), indicating ~5% lower odds of myopia per 0.1-unit increase in NDVI; cohort studies yielded a pooled OR of 0.91, with zone-specific pooled estimates showing a stronger effect within school grounds (*OR* = 0.96, 95% CI 0.94–0.98) and within a 500-m buffer (ORs around 0.90–0.91 across analyses) than at larger (1,000-m) scales. Heterogeneity was modest in cross-sectional syntheses (*I*^2^ = 37.3%) but substantial among cohort studies (*I*^2^ = 88.9%), indicating that study design and exposure metric choices materially influence pooled estimates. Accordingly, these quantitative results serve as the conceptual basis for the subsequent discussion, informing our examination of biological mechanisms, clarifying how variation in green-space metrics contributes to study heterogeneity, and shaping the public-health and urban-planning implications of promoting greener school and residential environments.

The findings of this review demonstrate a consistent inverse association between green-space exposure and myopia risk in children and adolescents, with the strongest protective effects observed in school environments and their immediate surroundings. This spatially structured pattern provides important clues to the underlying mechanisms. Children spend the majority of their waking hours in and around school, and greenness in these proximal environments likely contributes to repeated and cumulative outdoor light exposure throughout the day. So, improvements to on-campus greenery likely produce greater cumulative reductions in near-work exposure than distal greening. High-intensity natural light is known to stimulate retinal dopamine release, a key inhibitor of excessive axial elongation, partly by enhancing dopamine synthesis and signaling pathways that stabilize ocular growth and prevent the eye from elongating to inappropriate focal planes (40, 41). Prolonged outdoor light exposure may also help prevent myopia by slowing the excessive growth of the eye axis (41, 42). The fact that the protective association was most evident within the 500-meter buffer around schools aligns closely with the biological requirement for frequent, sustained light exposure to exert meaningful influence on ocular growth. In this sense, the spatial dependence observed in our synthesis is not incidental but reflects the necessity for greenness to coincide with children's actual daily activity spaces in order to produce measurable physiological effects. Behavioral pathways further reinforce these biological mechanisms. The connection between green spaces and myopia may be due to increased outdoor activity time and reduced screen time, both of which contribute to a lower risk of myopia (37, 43). Green environments tend to promote outdoor play (44), active commuting, and intermittent breaks that naturally reduce continuous near-work and screen-based activities (45). The longer the physical activity duration, the lower the probability of developing myopia (44). Such behavioral shifts increase opportunities for distance viewing and accommodation changes, which help maintain ciliary muscle function and reduce the risk associated with prolonged near focusing (42, 43). The modifying effect of screen time observed in several included studies—where the protective influence of greenness was weakened among children with high screen exposure—supports the interpretation that green-space benefits are partly realized through behavioral substitution. In other words, greenness is not only a passive environmental attribute but also a facilitator of healthier visual behaviors; where these behaviors are constrained, the protective effect becomes less pronounced.

Some of the heterogeneity across studies may be attributable to contextual or environmental co-factors. In dense urban settings, for example, greener neighborhoods often coincide with lower levels of air pollutants such as nitrogen dioxide and fine particulate matter, which have been implicated in retinal inflammation and oxidative stress (46–48). Though few primary studies measured air quality directly, this pathway remains a plausible contributor to stronger associations in heavily urbanized contexts. Landscape characteristics such as canopy structure and visibility may also influence how children interact with outdoor environments (44, 49), affecting opportunities for distance viewing and contributing to variation across different greenness metrics. Additional indirect pathways—such as circadian rhythm stabilization, whereby insufficient melatonin secretion may impair normal refractive development (17, 50) and reductions in psychological stress (51), which may otherwise induce extraocular muscular tension and contribute to ocular shape deformation (19) —may also play supplementary roles, although current evidence indicates that these mechanisms are secondary to the dominant influences of light exposure and visual-behavior patterns. Taken together, the evidence indicates that the protective effect of green spaces on myopia is most convincingly explained by a combination of cumulative light exposure and behaviorally mediated reductions in near-work burden, both of which are maximized when greenness is embedded directly within children's daily activity spaces. Other mechanisms may contribute in specific settings, but their influence appears context dependent and generally weaker. The convergence of biological plausibility, spatial coherence, and behavioral evidence offers a clear interpretive framework for the findings of this review and underscores the value of integrating greening strategies into school environments as a practical and potentially impactful approach to myopia prevention.

While the stronger protective associations observed for greenness within school grounds and the 500-m buffer suggest that children's daily exposure patterns play a critical role, green-space exposure and outdoor time represent related but distinct pathways. Theoretically speaking, contact with green space can affect eyesight in at least two completely different ways: one is to increase children's time outdoors (behavioral adjustment), and the other is to change the physical environment (such as improving the intensity of the surrounding light, changing the spectral composition, reducing local air pollution or changing the clues of visual distance). Randomized trials have confirmed that increasing outdoor time can directly reduce the incidence of myopia, but our research results show that children's outdoor activities sites, especially in greener and closer to schools, may enhance or change this effect. Greenness may operate partly by increasing children's likelihood of being outdoors, but also through environmental mechanisms not captured by time-use alone, such as higher ambient light levels, more open visual distance cues, and improved local air quality. To better separate these pathways, future studies should jointly measure area-level greenness and individual-level outdoor time (preferably with objective tools such as wearable light or GPS sensors) to enable formal mediation analyses. Studies that adjust for outdoor time can further clarify whether greenness contributes benefits independent of behavior.

The subgroup results indicated that NDVI was inversely associated with myopia prevalence, with the strongest effects observed for greenness within school boundaries and the surrounding 500-meter buffer. This spatial pattern aligns closely with children's daily visual exposure: because students spend most of their waking hours at school, proximal greenness constitutes the portion of the environment they encounter most frequently (52). Such repeated exposure increases opportunities for distance viewing, ambient light stimulation, and visual breaks from near work—mechanisms directly supported by the biological and behavioral pathways identified in this study. The greater effect observed for the 500-meter buffer, compared with the 1,000-meter buffer, further reflects children's limited independent mobility (53). Green spaces within several hundred meters are far more likely to be integrated into routine routes between home and school and to remain visible from classrooms, thereby exerting sustained influence on the visual environment. This exposure pattern provides a mechanism-driven explanation for why proximal greenness showed stronger associations in our meta-analysis, rather than suggesting that all green spaces are equally protective. Although previous studies have highlighted the relevance of school-adjacent greenery, the empirical rationale has been limited. Our findings strengthen this evidence by demonstrating that protective effects are concentrated within children's actual activity spaces, underscoring that the “effective dose” of green-space exposure is determined not only by quantity but by spatial proximity and daily visibility. However, NDVI is an indirect and imperfect proxy for ocular light exposure. As an area-level indicator of vegetation density, NDVI does not account for individual behavioral patterns, such as time spent outdoors, activity type, or actual light intensity reaching the retina. Moreover, factors such as seasonality, urban morphology, shading, and indoor–outdoor transitions may substantially modify the relationship between surrounding greenness and true ocular light exposure. Consequently, associations based on NDVI may partially reflect broader environmental or contextual influences rather than direct biological effects of light exposure *per se*. Given the limited research on optimal buffer sizes and the absence of consistent evidence for nonlinear exposure–response relationships, further studies are needed to evaluate dose thresholds and refine spatial planning strategies. Some predefined subgroups contained only a single study, which limits their utility for formally exploring sources of heterogeneity. In such cases, subgroup-specific estimates were presented for descriptive purposes only and should not be interpreted as evidence of differential effects across study characteristics. This limitation reflects the current scarcity of studies using comparable exposure definitions and study designs, particularly among cohort studies, and underscores the need for additional well-powered and methodologically harmonized research to enable more robust subgroup and meta-regression analyses. Nevertheless, the present results suggest that enhancing greenness in and immediately around schools may offer the most impactful environmental approach for reducing myopia risk.

The substantial heterogeneity observed in this meta-analysis (*I*^2^ = 88.9%) likely reflects genuine methodological and contextual diversity across studies rather than statistical noise. Variations in exposure quantification—ranging from NDVI and SAVI to proportional green area, shadow-based visible greenness, and park-area metrics—capture different ecological and visual attributes of green space and, when combined with heterogeneous buffer definitions (school boundaries to 1,000 m), naturally generate divergent effect estimates. Outcome measurements also varied widely, including cycloplegic and non-cycloplegic autorefractors, visual acuity charts, self-reported vision, and spectacle use, each contributing differing levels of precision and thereby increasing between-study variance. Additional heterogeneity stems from differences in study design (cohort vs. cross-sectional), age distributions, urbanicity, and inconsistent adjustment for key behavioral and environmental factors such as outdoor activity, screen time, socioeconomic status, and air pollution. Remote-sensing differences (season, resolution, and processing algorithms) and diverse statistical modeling choices further amplify variability. To evaluate whether age heterogeneity was a major contributor, we conducted a sensitivity analysis excluding the largest study [(36); *N* ≈ 1,245,000; ages 8–15] (Supplementary Figure S2). The pooled NDVI–myopia association remained virtually unchanged (pooled *OR* ≈ 0.90; 95% CI: 0.90–0.91; *p* < 0.001), and heterogeneity remained high (*I*^2^ = 92.7%), indicating that age variation across studies reflects expected developmental differences rather than methodological bias and is not a primary driver of heterogeneity. Because the limited number of cohort studies precluded reliable subgroup analyses or meta-regression, some sources of between-study heterogeneity could not be formally quantified, which may have reduced the precision of the pooled estimates. Nevertheless, the direction and statistical significance of the associations were consistent across study designs, suggesting that the overall findings are unlikely to be driven by a single study or methodological artifact. Collectively, these findings suggest that the high *I*^2^ arises from substantive variations in exposure definitions, measurement precision, contextual environments, and covariate control. Consequently, the pooled estimate should be interpreted as an average effect across diverse real-world conditions, while more consistent patterns—particularly the robust associations for NDVI-based metrics and greenness within school-proximal 500 m buffers—likely represent the most reliable and policy-relevant evidence. Future work utilizing harmonized exposure measures, standardized refractive assessments, and consistent covariate adjustment will be essential for reducing heterogeneity and improving causal inference.

Beyond the primary effects of greenness, this study found that several covariates modified the association between green-space exposure and myopia risk. The stronger protective effect observed among girls in some studies (54, 55)may reflect biological susceptibility related to pubertal hormonal regulation, which can accelerate ocular growth and heighten myopia risk (56). Under such vulnerability, increased exposure to green environments may offer relatively greater benefits by providing more frequent outdoor light exposure and visual breaks. Socioeconomic status (SES) also influenced the association. Children in lower SES areas—where access to healthcare and high-quality green spaces is often limited—showed higher baseline myopia risk, suggesting that even modest improvements in local greenness may confer disproportionate protective effects in these communities (57, 58). Screen time further modified the green space–myopia relationship. Studies (59) showing that higher NDVI was associated with reduced sedentary behavior and lower screen use support the interpretation that green environments protect vision partly by displacing indoor near-work activities (60, 61). This aligns with established evidence that prolonged screen exposure reduces outdoor time and light exposure, thereby increasing myopia risk. Together, these findings indicate that the benefits of greenness are shaped by biological, social, and behavioral contexts. Identifying groups with heightened vulnerability—such as girls, children from low-SES backgrounds, and those with high screen-time patterns—may help optimize the targeting and effectiveness of greening strategies for myopia prevention.

Although the magnitude of the observed association between NDVI and myopia risk appears modest at the individual level (approximately a 5% reduction in odds per 0.1-unit increase in NDVI), its public-health relevance should not be underestimated. Given the extremely high prevalence of myopia among children and adolescents—particularly in East Asia—even small relative risk reductions may translate into substantial absolute reductions in case numbers at the population level. Importantly, our findings indicate stronger protective associations in school-based environments, especially within school campuses and proximal 500-m buffers, suggesting that targeted greening strategies in these settings may yield measurable benefits for visual development. Environmental greening represents a structural, low-cost, and sustainable intervention that operates independently of individual behavioral compliance, in contrast to approaches focused solely on reducing screen time or prescribing outdoor activity. From a population-health perspective, modest effect sizes associated with ubiquitous environmental exposures can generate meaningful long-term benefits when implemented at scale, supporting the prioritization of school-centered and neighborhood greening initiatives to help mitigate myopia risk among children and adolescents. Based on these findings, relevant government departments, schools, and families should take measures to increase the availability of green spaces accessible to children and adolescents, thereby reducing the risk of myopia. Government authorities should emphasize the importance of integrating green spaces into urban development, support urban greening, and improve the greenery around schools and homes through tree planting. Higher levels of green space in campuses can offer additional health benefits, such as improving mental state and mood, enhancing mental health and cognitive function (62–64), and reducing the incidence of cardiovascular diseases and diabetes (65). At the same time, potential negative impacts of urban greening should also be considered, such as the increased incidence of allergic diseases due to excessive biodiversity (16), and factors related to vegetation distribution (e.g., the aggregation and connectivity of green spaces, which are linked to higher myopia rates) (30), to optimize health benefits. Schools and parents should encourage children and adolescents to engage in sufficient outdoor activities in green spaces and reduce screen time. Future research should also explore the potential moderating effects of outdoor time and screen time on the relationship between green spaces and the risk of myopia in children and adolescents, and investigate the thresholds and variations in these moderating effects.

The strengths of this study lie in the fact that all the included studies are cohort and cross-sectional studies, providing strong causal inference. Additionally, the quality of the studies included in the meta-analysis is high (8 high-quality studies and 3 medium-quality studies). Furthermore, this study considered the exposure of children and adolescents to green spaces around schools and homes, with a large sample size that is representative of children and adolescents in China. Most of the vision data from the samples were objectively measured using clinically validated and standardized protocols, ensuring the data's reliability and validity to the greatest extent. However, there are several limitations in this study: (1) Literature retrieval was limited to English and Chinese, which may lead to language or cultural bias. (2) Due to the small sample size of cohort studies included in the meta-analysis, sensitivity analysis could not be performed, making it impossible to fully assess the robustness of the results, thus limiting the relevance of the conclusions. (3) Although NDVI provides an objective measure of greenness, it does not capture how or whether individuals actually utilize the available green spaces. (4) Some data on covariates and mediators were collected through surveys to obtain subjective outcome indicators, which could introduce bias into the results. (5) The green space indicators used in this study were only assessed through remote satellite imagery, and the different indices of green space exposure created by different data collection methods may introduce content bias. (6) The generalizability of our findings is limited by the geographical concentration of the included studies, with the evidence base predominantly derived from China. This may restrict the applicability of our conclusions to other regions with differing environmental profiles, urbanization patterns, and genetic backgrounds. (7) Due to the lack of stratified results by age group, the potential differences in myopia risk, exposure to green spaces, and significance for different age groups were not explored in-depth. (8) Although publication bias was assessed using funnel plots and Egger's regression test, the limited number of studies in several analyses restricted the statistical power of these assessments. Evidence of small-study effects was observed in the cross-sectional studies, and therefore publication bias cannot be fully excluded. These limitations warrant further research to address and explore.

## 5 Conclusions

Systematic reviews and meta-analyses indicate that increased exposure to green spaces has a positive effect on reducing the risk of myopia in children and adolescents. A 0.1 increase in NDVI is associated with a decrease in the myopia prevalence among children and adolescents. Moreover, the effectiveness of myopia risk prevention is particularly pronounced within school grounds and in the 500m buffer zone around schools. Therefore, schools and government departments should place greater emphasis on enhancing vegetation coverage around school pathways and students' activity areas to provide more environmental support for myopia prevention in children and adolescents. However, given the substantial heterogeneity observed across studies, particularly in cohort analyses, these findings should be interpreted cautiously.

## Data Availability

The original contributions presented in the study are included in the article/Supplementary material, further inquiries can be directed to the corresponding authors.
