# Supplementary material for: The relationship between green space and myopia in children and adolescents: a systematic review and meta-analysis
Source: Front Public Health. 2026 Apr 1;14:1712259. doi: 10.3389/fpubh.2026.1712259 (PMC13079651; doi:10.3389/fpubh.2026.1712259)
Supplement: Supplementary file 1 [file Data_Sheet_1.pdf]

Supplementary Material

1 Supplementary Figures and Tables

	95%Cl	P
**Cross-sectional studies**	-2.565 to -0.293	**0.025***
**Cohort studies**	-1.593 to 6.386	0.192

Supplementary Tables S1. Egger's regression tests

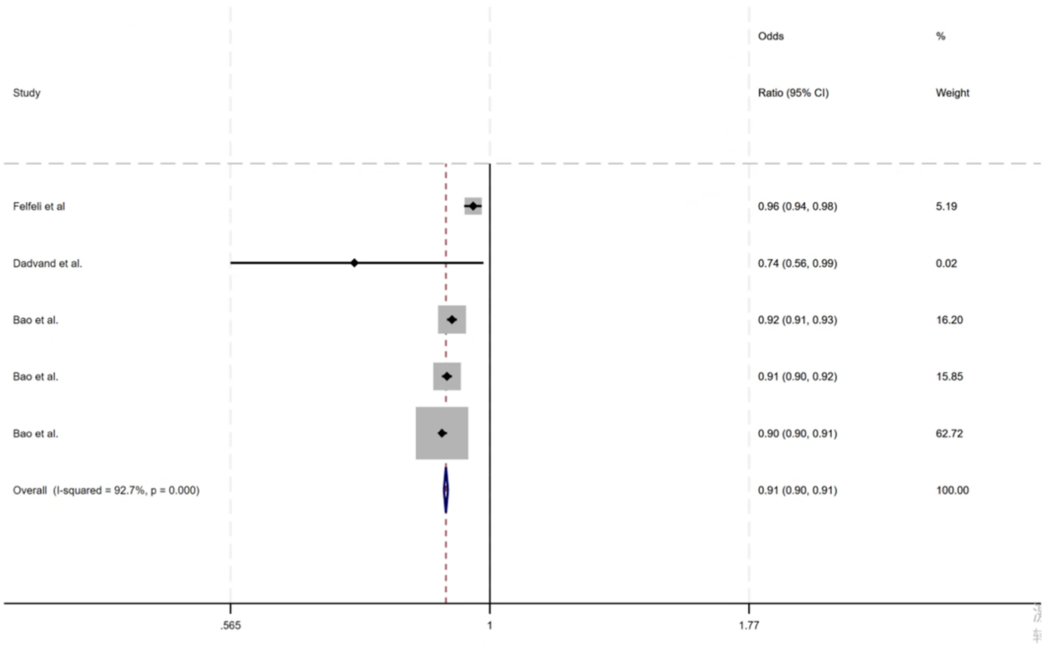

Supplementary Figure S2. Sensitivity Analysis of the Association between Green Space and the Incidence of Myopia in Children and Adolescents, by Age Group
